# Home-based neuro-cardiac rehabilitation in congenital heart disease: the QUALINEURO-REHAB trial study protocol

**DOI:** 10.1136/bmjopen-2026-120953

**Published:** 2026-10-05

**Authors:** Johanna Calderon, Sylvie Martins, Alfred Hager, Lauriane Cornuault, Cecile Jore, Mathieu Andrianoely, Marie-Christine Picot, Florence Francis, Florence Saillour, Marion Kret, Anne Cécile Huby, Marion Audie, Pierre-Marie Duboue, Jelena Hubrechts, Gregoire De La Villeon, Marie Vincenti, Gaelle Marguin, Gwladys Desmure, Julia Hock, David Schmitz, Damien Bonnet, Stephane Moniotte, Sophie Guillaumont, Pascal Amedro, on behalf of the QUALINEURO-REHAB study group, Fanny Bajolle

**Affiliations:** 1 Cardiac Neurodevelopmental Center - CNC Necker, National Reference Center for Complex Congenital Heart Diseases M3C, APHP, Université Paris Cité, Hôpital Necker-Enfants Malades, Paris, France; 2 French National Institute of Health and Medical Research, PhyMedExp, Cardiac Neurodevelopmental Research Group, Inserm U1046, Montpellier, France; 3 Unit of Pediatric and Congenital Cardiology, Montpellier University Hospital, Montpellier, France; 4 Department of Pediatric and Congenital Cardiology, National Reference Centre for Complex Congenital Heart Disease, Bordeaux, France; 5 Department of Congenital Heart Defects and Pediatric Cardiology, TUM University Hospital, Deutsches Herzzentrum München, Munich, Germany; 6 INSERM U1045, University of Bordeaux, IHU Liryc Institut des Maladies du Rythme Cardiaque, Pessac, France; 7 Epidemiology and Clinical Research Department, Montpellier University Hospital, Montpellier, France; 8 Public Health Department, Bordeaux University Hospital, Bordeaux, France; 9 Public Health Department, Medical Information Unit, CHU Bordeaux, Bordeaux, France; 10 Bordeaux Population Health, INSERM U1219, Bordeaux, France; 11 USMR, CHU de Bordeaux, Bordeaux, France; 12 Pediatric cardiology, University Hospital Centre Bordeaux, Pessac, France; 13 Le Hillot Cardiac Rehabilitation Center, Bordeaux, France; 14 Division of Pediatric Cardiology, Department of Pediatrics, Cliniques universitaires Saint-Luc, Brussels, Belgium; 15 UCLouvain, Louvain-la-Neuve, Belgium; 16 Montpellier University Hospital, Montpellier, France; 17 Pediatric Cardiology, University Hospital of Montpellier, Montpellier, France; 18 U1046 PhyMedExp, National Institute of Health and Medical Research, Montpellier, France; 19 Patient Association Petit Coeur de Beurre, La Garenne Colombes, France; 20 Patient Association Petit Coeur de Beurre, Paris, France; 21 Department of Congenital Heart Defects and Pediatric Cardiology, Deutsches Herzzentrum München, Munich, Germany; 22 TUM University Hospital, Munich, Germany; 23 Clinical Research and Innovation Department, Bordeaux University Hospital, Bordeaux, France; 24 M3C Cardiologie Pediatrique, Hôpital Universitaire Necker-Enfants Malades, Paris, France; 25 University Hospital of Montpellier, Montpellier, France; 26 Department of Pediatric and Congenital Cardiology, George Washington University School of Medicine & Health Sciences, Heart & Lung Center — Children’s National Hospital, Washington, DC, USA; 27 University of Bordeaux, Talence, France

**Keywords:** Congenital heart disease, Developmental neurology & neurodisability, Patient Reported Outcome Measures, Physical Fitness, Psychosocial Intervention

## Abstract

**Introduction:**

Neurodevelopmental impairments and physical deconditioning are the most common long-term morbidities in congenital heart disease (CHD). Holistic interventions including combined neuro-cardiac training may improve physical and mental health in youth with CHD.

**Methods and analysis:**

This is a European multicentre, two-arm randomised controlled study to test the efficacy of a home-based neuro-cardiac rehabilitation programme including physical activity and neuropsychological training versus the standard of care in children, adolescents and young adults with complex CHD. Patients aged 8–25 years who underwent infant surgical and/or catheter-based cardiac interventions will be randomly allocated to either an intervention group including a 12-week home-based cardiovascular rehabilitation and executive function training or to a control group. All participants will undergo a baseline assessment and a 12-month follow-up. The primary outcome is the difference in change in the Pediatric Quality of Life Inventory self-reported health-related quality of life score between baseline and final assessment for the intervention versus the control group. Secondary outcomes include standardised neuropsychological measures of executive function, social cognition and mental health. Cardiovascular secondary outcomes include cardiopulmonary fitness parameters such as maximum oxygen uptake, muscular strength and level of physical activity. Efficacy of the intervention will be evaluated by comparing within-subject group differences from baseline to 12-month follow-up, using an intention-to-treat analysis.

**Ethics and dissemination:**

The trial protocol was first approved by the sponsor’s drawn National Ethics Committee (CPP Paris-III, number 2023-A00038-37) and registered on Clinicaltrial.gov (number NCT05670132). It was also approved by Ethics Committees in Germany (Technische Universitat Munchen, number 2023-276-S-KH) and Belgium (UCLouvain, number B4032025000116).

**Trial registration number:**

NCT05670132.

**STRENGTHS AND LIMITATIONS OF THIS STUDY**

- This is a European multicentre, two-arm randomised controlled study to test the efficacy of a home-based neuro-cardiac rehabilitation programme including physical activity and neuropsychological training vs the standard of care in children, adolescents and young adults with complex congenital heart disease (CHD).
- All participants will undergo a baseline assessment and a 12-month follow-up, with measures of quality of life, neurodevelopmental outcomes and cardiovascular indicators.
- A holistic intervention for two of the most frequent clinical concerns in CHD (neurodevelopmental and cardiorespiratory limitations) will be tested in three European countries (France, Belgium and Germany).
- Efficacy of the intervention will be evaluated by comparing within-subject group differences from baseline to 12-month follow-up, using an intention-to-treat analysis.
- Participants’ longer-term outcomes may be re-evaluated in terms of cognitive, behavioural and socio-emotional improvements following the intervention.

## Introduction

 Major advances in paediatric cardiology, including prenatal screening of congenital heart disease (CHD), infant cardiac surgery and interventional catheterisation, have increased the survival of children with CHD. Today, more than 90% of these patients reach adulthood.^1^ The increase in the population of survivors of complex CHD has been accompanied by an awareness of adverse long-term sequelae. In the last decade, two major morbidities have been recognised as the most prevalent issues in CHD: neurodevelopmental issues and physical deconditioning.^2–6^ Studies conducted in several countries have shown that neurodevelopmental disorders and reduced exercise capacity constitute a critical public health priority in CHD.^7^ In 2024, updated American Heart Association recommendations for the neurodevelopmental assessment and management of patients with CHD emphasised the need to develop not only screening and diagnostic plans but also interventional strategies that can prevent or mitigate these morbidities.^7 8^

Neurodevelopmental impairments and mental health disorders, including executive function issues, represent a major concern in CHD.^9 10^ Executive function impairments have been consistently reported in children,^11^ adolescents^12^ and adults with CHD.^13^ These can include difficulties with self-regulation, working memory and goal-directed behaviour. Executive function impairments carry implications for the educational achievement, future employment and quality of life of individuals with CHD. These challenges are present from childhood throughout adulthood and, if untreated, they may predispose individuals to later addiction, obesity and risk-taking behaviours.^14 15^ Dysexecutive symptoms can also affect decision-making and health-related behaviours such as engaging in physical activity to promote cardiovascular health.

Early onset of physical deconditioning is commonly observed in youth with CHD,^3^ even after successful cardiac surgical repair and despite the absence of any sports restriction.^16^ Indeed, maximum oxygen uptake (VO_2max_), for example*,* the main marker of cardiopulmonary fitness, decreases faster in CHD than in healthy children, resulting in impaired health-related quality of life (HRQoL) and increased cardiovascular risk.^2 17^ Cardiopulmonary fitness impairment in children with CHD is multifactorial, cumulating reduced aerobic capacity inherent to the cardiac condition, impaired muscle and lung function, and behavioural barriers to physical activity.^18 19^ Yet, physical activity has critical benefits for cardiac and mental health.^20 21^ Most children with CHD should follow the WHO guidelines and cumulate at least 60 min of daily moderate to vigorous physical activity.^22^ A sedentary lifestyle and its consequences in youth, especially those with pre-existing cardiac conditions, is a major public health issue.^23–25^ Currently, no structured preventive rehabilitation programme has been developed to address both neuropsychological and physical impairments in patients with CHD.^26–28^

Our previous work has shown that some executive functions such as inhibitory control can be amenable to improvement following intensive cognitive computerised training.^28^ However, generalisation to broader neuropsychological areas was not achieved by cognitive training alone, suggesting that a holistic integrative approach is needed. Recently, our group demonstrated the positive and long-lasting effect of an early hybrid cardiac rehabilitation programme on HRQoL, body mass index, disease knowledge and physical activity, in youth with CHD.^26^ However, the VO_2max_ increase observed at the end of cardiac rehabilitation (+3 mL/kg/min) had no lasting effect, probably due to the moderate-intensity nature of the exercise training programme. Importantly, no active neuropsychological component was included in this study.

Indeed, a home-based ‘neuro-cardiac’ rehabilitation programme, including neurocognitive training and neuropsychological guidance as well as innovative adapted physical activity programme with high-intensity interval training, may have greater potential for positive effects in CHD. Such intervention should include key aspects of rehabilitation tailored to this population, with a holistic approach based on physical and neuropsychological prevention. In the QUALINEURO-REHAB multicentre randomised controlled trial, we aim to assess the impact of a home-based neuro-cardiac rehabilitation programme on HRQoL, physical health, neuropsychological and mental health outcomes in children, adolescents and young adults with complex CHD.

## Methods

### Study design

The QUALINEURO-REHAB trial is a prospective randomised controlled multicentre two-arm parallel efficacy study with a 12-week intervention and a final assessment at 12-month follow-up. Participants will be recruited from six European tertiary care hospital centres in France, Belgium and Germany over a period of 24-month inclusion period. Eligible subjects will be identified from each hospital centre database and informed about the study during routine outpatient follow-up.

Screening procedures may be performed up to 42 days before enrolment to accommodate routine clinical practice across participating centres, where the cardiology consultation, physical examination, ECG, echocardiography and cardiopulmonary exercise test (CPET) are not always scheduled on the same day. These routine clinical investigations are reviewed to confirm eligibility and ensure safe participation in the physical rehabilitation component of the intervention.

Following enrolment, all participants undergo standardised baseline study assessment before randomisation, including physical and neuropsychological evaluations. The same study assessments are repeated at the 12-month follow-up visit. Randomisation is performed after completion of the baseline assessment.

Participants allocated to the intervention group receive a 12-week home-based neuro-cardiac rehabilitation programme. After completion of the intervention, they return to usual clinical follow-up until the 12-month assessment, with no additional supervised study intervention. Participants allocated to the control group receive standard care throughout the study period. No crossover to the intervention is planned after study completion.

The study flowchart is presented in figure 1. Assessments and visit timeframes are detailed in table 1.

**Figure 1:**
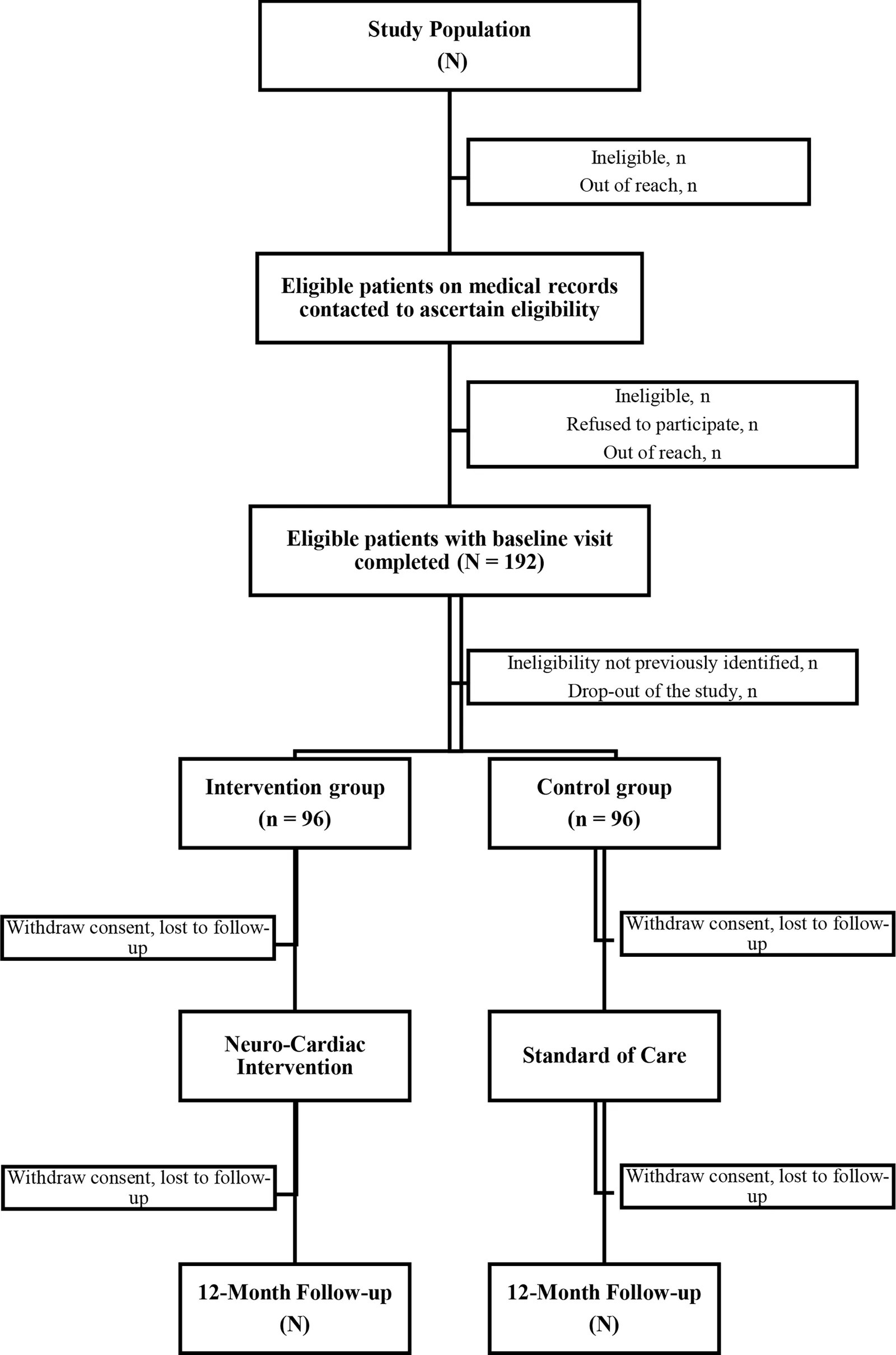
Flowchart of trial design.

**Table 1:** Assessments and visit timeframes

Assessments and visit timeframe		All patients			Intervention group only			All patients
		Screening (D42 to D0)*	Inclusion visit D0	Randomisation D7 (+ 7 days)	Initiation visit (week 1) (D14 to D49)	Home-based neuro-cardiac rehabilitation (week 1 to week 12 (+2 weeks))	End of neuro-cardiac rehabilitation D126±30 days	End of study D365±30 days
		Visit 0	Visit 1		Visit 2		Visit 3	Visit 4
Patient information		x†	x†					
Verification of eligibility criteria		x	x		x			
Patient consent signature			x†					
Consultation with congenital cardiologist (investigator)		X†						X†‡
Physical examination		X†						X†‡
Echocardiography		X†						X†‡
ECG		X†						X†‡
Cardiopulmonary exercise test (CPET)		X†						X†‡
Socio-demographic data			x†					
Research outcomes assessment	PedsQL health-related quality of life self-questionnaire (and proxy questionnaire in minors)		x†					x†
	Neuropsychological assessment§		x†					x†‡
	Upper limb muscle strength: Handgrip test		x†					x†‡
	Physical activity level: ActiGraph GT3X accelerometer		x^a,c^					x†‡¶
	Physical activity level: PAQ-C, PAQ-A or IPAQ questionnaires		x†					x†‡
Randomisation				x**				
Consultation with sports physician					x††‡‡	x‡§§	X††¶¶	
Adverse event collection					x	x	x	x
Consultation with health educator					x††	x††§§	x††	
Intervention by adapted physical activity (APA) trainer					x††***	x††***	x††***	
Consultation with neuropsychologist					x††	x††	x††	
Neuro-cardiac programme					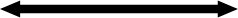			

D, day; X, routine follow-up visit/outcome assessment; x, research visit/outcome assessment.

* The screening period extends up to 42 days before inclusion to accommodate routine clinical practice across participating centers, where the cardiology consultation, ECG, echocardiography, and cardiopulmonary exercise test may be scheduled over several visits because of local logistical constraints.

† On-site at the hospital.

‡ It will be possible to carry out this visit in two stages (cardiological evaluation and neuropsychological assessment) over a period of 8 days. The quality-of-life questionnaire, which is the main assessment criterion, should be completed first, before the neuropsychological assessment.

§ Behavior Rating Inventory of Executive Function (BRIEF) scale score or BRIEF-A for participants older than 18 years, Wisconsin Card Sorting Test for children and adults, Score on the main indices of the Wechsler Intelligence Scale for Children (WISC-V) or the Wechsler Adult Intelligence Scale (WAIS-IV), NEPSY-II scale scores, Score from visual and verbal Wechsler Adult Memory Scale, Vineland-2 proxy questionnaire, Score of the Multiscore Depression Inventory for Children (MDI-C) or Beck Depression Inventory (BDI-2) for participants 17 years and older, Revised Children’s Manifest Anxiety Scale (R-CMAS) score or STAI Anxiety Questionnaire for participants 17 years and older, Resilience Scale for Children (RS10) score or Resilience Scale for Adults.

¶ Worn 7 days after inclusion (D0) and 7 days before the end of the study (12-month follow-up, D365).

** After validation of neuropsychological tests.

†† Videoconference.

‡‡ This remote consultation with the sports physician corresponds to the intervention initiation visit during week 1 and is scheduled as the first visit of the initiation week, before the rehabilitation sessions begin.

§§ Videoconference with the sports physician and/or health educator could be realised during the neuro-cardiac programme, if necessary, at the request of the patient (and/or legal guardian), or investigator.

¶¶ This remote consultation with the sports physician corresponds to the intervention exit visit at the end of week 12 and is scheduled as the last visit of final intervention week, after completion of the rehabilitation sessions.

*** At the patient’s home. V0 and V1 can be merged if all screening examinations are routinely organised on the same day by the CHD center, provided that (1) the patient and/or his/her legal guardians have had a period of reflection between information and consent to participate in the research, and (2) all baseline examinations could be technically organised on the same day too.

CHD, congenital heart disease; PAQ-A, Physical Activity Questionnaire for Adolocents; PAQ-C, Physical Activity Questionnaire for Children; PedsQL, Pediatric Quality of Life Inventory.

### Study population

Patients aged 8–25 years with a CHD of moderate-to-great complexity^29^ and a history of cardiac surgery and/or catheter-based intervention(s) during the first year of life will be eligible for this study. Patients with severe cardiac, haemodynamic, neurological or psychiatric conditions, inability to follow instructions and/or answer questionnaires, absolute contraindications for CPET, inability to undergo the neuro-cardiac intervention or recent or planned medical or surgical intervention will not be eligible for the study. A complete list of inclusion and exclusion criteria is provided in box 1.

Box 1 **Eligibility criteria**

**Inclusion criteria**

- Patient with a CHD of moderate to great complexity as defined in the 2019 AHA/ACC guidelines.^29^
- History of cardiac surgery and/or catheter-based cardiac intervention(s) during the first year of life.
- Age between 8 and 25 years at the time of enrolment.
- Parental or legal guardian’s consent for minors (< 18 years) and personal consent for adults.

**Exclusion criteria**

- Unstable and/or severe heart failure: severe heart failure (class IV NYHA functional class), recent decompensated heart failure requiring hospitalisation and/or any significant change in medication (<3 months before enrolment), systolic ventricle dysfunction (left ventricle or systemic ventricle ejection fraction <50%).
- Severe hypoxemia: pulse oxygen saturation (SpO2) at rest <85%, and/or SpO2 at exercise <80%, and/or patient requiring oxygen therapy.
- Pulmonary hypertension as defined by the 2020 ESC guidelines,^64^ whatever the aetiology.
- Significant systolic right ventricle hypertension (systolic right ventricle pressure >50% of systemic systolic pressure).
- Uncontrolled arrhythmia: symptomatic treated or untreated arrhythmia at rest and/or exercise, treated arrhythmia with sustained supraventricular or ventricular tachycardia on ECG monitoring or during exercise and/or cardiopulmonary exercise test (CPET), occurrence or aggravation of any supraventricular or ventricular arrhythmia during exercise and/or CPET.
- Advanced atrioventricular block (degree 2 or 3), occurrence or aggravation of any conduction disorder during exercise and/or CPET.
- Uncontrolled arterial hypertension at rest (eg, if the blood pressure at rest during the consultation >140/90 mm Hg in adults, >95th percentile in children).
- Acute or recent (<3 months) myocarditis and pericarditis.
- Symptomatic and/or significant aortic or subaortic stenosis (maximal gradient >50 mm Hg).
- Non-corrected coarctation of the aorta (surgical or catheter-based repair) with a clinical systolic gradient >20 mm Hg.
- Dilatation of the aorta (aortic root >40 mm in adults, >2 Z-score in children), except in repaired congenital heart disease with dilatation of the aorta inherent in the malformation and without significant risk of aortic dissection (tetralogy of Fallot, pulmonary atresia with ventricular septal defect, truncus arteriosus, transposition of the great arteries, single ventricle, double outlet right ventricle).
- Severe hypertrophic obstructive cardiomyopathy.
- Acute systemic illness.
- Recent (<3 months) intracardiac thrombus, embolism or thrombophlebitis.
- Inability to follow instructions and/or complete the questionnaires, as determined by the investigator.
- Absolute contraindications for CPET: fever, uncontrolled asthma, respiratory failure, acute myocarditis or pericarditis, uncontrolled arrhythmias causing symptoms or haemodynamic compromise, uncontrolled heart failure, acute pulmonary embolus or pulmonary infarction, and patients with mental impairment leading to inability to cooperate.
- Inability to undergo the physical intervention: inability to physically exercise, any invasive medical intervention occurring within 6 months preceding the enrolment or scheduled during the 12-month study period (such as cardiac surgery, catheter-based intervention, orthopaedic surgery, chemotherapy for cancer, or any other significant medical treatment or intervention, as determined by the investigator).
- Patient with a previously diagnosed or suspected severe psychiatric disorder (major depression with or without suicidal ideation, psychotic diagnoses, severe anxiety/panic disorders) requiring hospitalisation and/or continuous specialised care by a psychiatrist, as determined by the investigator.
- Patients who benefited from a cardiac rehabilitation within the 12 months preceding the enrolment.
- Patients who benefited from a computerised executive function or attention training such as Cogmed Working Memory Programme within the 12 months preceding the enrolment.
- For female patients: pregnancy, pregnancy plan or breastfeeding woman following questioning of the patient.
- Patients deprived of liberty due to an ongoing legal procedure, adult’s patient under guardianship or curatorship or unable to personally express their consent.
- Any other clinical and/or pharmacological treatment that is believed to interfere with the study or the optimal clinical care.
- Initiation or total withdrawal of psychotropic medication (eg, any psychotropic medication including methylphenidate, benzodiazepines, and mood stabilisers) within the 6 months preceding the enrolment.
- Patient participating or wishing to participate in any interventional clinical research (drug trial, medical device, non-drug trial).

### Trial procedures

Randomisation will be performed using a computer-generated assignment sequence, stratified by the country of the clinical recruitment site and the patient age group (8–12, 13–17 and 18–25 years old). Participants will be randomly allocated in a 1:1 ratio to either the intervention or the control group. The two groups will be (1) the QUALINEURO-REHAB intervention group, that is, participants in the neuro-cardiac rehabilitation programme, and (2) the control group, that is, participants following standard of care with no neuro-rehabilitation programme during the 12-month study period. All participants can continue treatments and medications that are already in place at the time of enrolment.

### Intervention: neuro-cardiac rehabilitation programme

The QUALINEURO-REHAB intervention is a 12-week home-based neuro-cardiac rehabilitation programme. Based on a holistic approach, the programme includes two components, that is*,* neuropsychological and cardiac rehabilitation, using a multidisciplinary secured digital health platform, and delivered by trained healthcare professionals: sports physicians (eg*,* paediatric and congenital cardiologists), neuropsychologists, health educators (eg*,* specialist nurses), and exercise physiologists or physical therapists trained in adapted physical activity (eg*,* adapted physical activity (APA) trainers). The same programme will be delivered in all investigating centres. Figure 2 presents all the components of the intervention, and table 1 summarises the intervention schedule and study visits, including the initiation visit (week 1), the 12-week intervention, the exit visit (week 12) and the final 12-month follow-up assessment.

**Figure 2:**
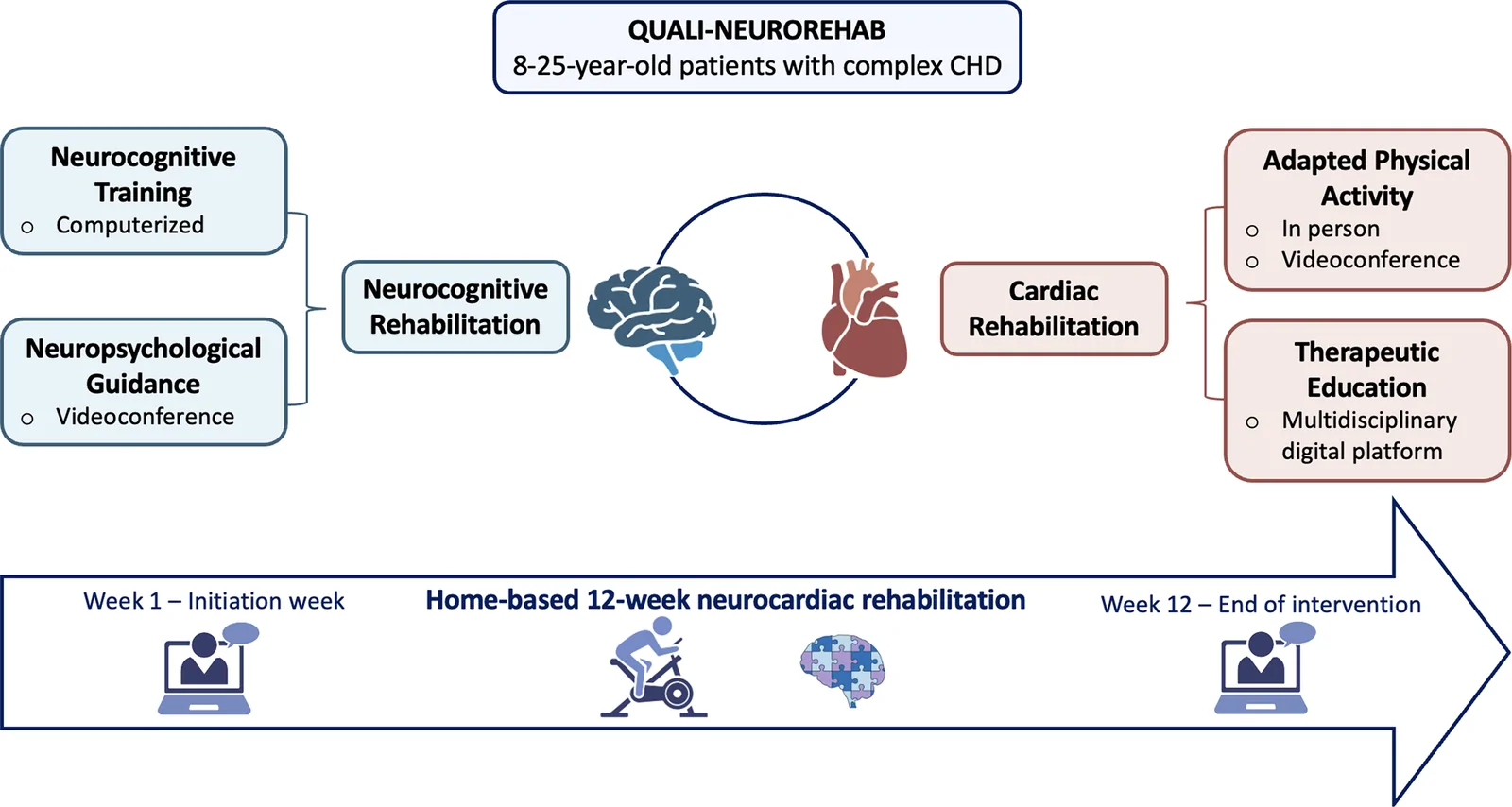
QUALINEURO-REHAB intervention. CHD, congenital heart disease.

The neuropsychological rehabilitation component includes neurocognitive training based on two weekly sessions of executive function training (25 min each, using the updated computerised Cogmed cognitive programme^30^ with a reward system) and weekly patient and parent neuropsychological 1-hour follow-up guidance sessions focused on executive functioning in daily life. Cogmed Working Memory programme has been chosen to train specific executive function skills, including inhibitory control (ie, the ability to control one’s impulsive responses and inhibit automatic tendencies), selective and sustained attention, and auditory and visual working memory. In this computerised programme, the participant is instructed to complete three exercises requiring recalling item spatial locations, numbers and letters backwards and sequences. All exercises are adapted to each participant’s level of executive functioning and encourage progression with a reward system (ie, visual and auditory encouragement, as well as earning ‘points’ to build a city at the end of each session). The neuropsychological follow-up is delivered to either the parent (for minor participants) or adult participants (at least 18 years old). The content and materials are adapted by age range (8–12 years, 13–17 years and 18 years and plus). Each individual session follows a predefined programme and content including sessions on ‘hot’ (emotional regulation) and ‘cold’ (attention, cognitive flexibility and planning) executive functions and practical activities at home to improve executive functioning. Participants will also bring their own life experiences and challenges to serve as starting points for neuropsychological guidance focusing on real-life executive functioning. Support neurocognitive booklets, specifically designed for this study, are provided by neuropsychologists to participants (parent and patient versions by age groups) to aid in guidance strategies to optimise executive functions (eg*,* planning, attention, control, emotional regulation, etc.).

The cardiac rehabilitation component includes an exercise training programme of two weekly sessions (1 hour each) and patient education support. For each participant, a single APA trainer will deliver the 24 exercise training sessions in accordance with the medical prescription and will complete a report for each session on the digital platform. One out of two weekly sessions will be delivered by the APA trainer at home and the other by videoconference on the digital platform. Each session includes 30 min of high-intensity interval training (HIIT) performed on a bicycle mounted on an indoor home trainer, followed by 30 min of individualised exercise training focused on muscle strengthening and functional activity. The intensity of the HIIT programme is individually prescribed from each participant’s baseline CPET, using heart rate targets, thereby accounting for individual exercise capacity and age-related physiological differences (online supplemental table 1). A bicycle, home trainer and smartwatch for heart rate monitoring are provided to each participant throughout the 12-week intervention. The patient education support will be supervised by the same health educator for each participant, involving two videoconferences (1 hour each) with the patient (or their parents for participants <18 years old) at programme entry and exit. The health educator will evaluate the patient’s educational needs, select appropriate educational e-content (document, drawing, photo, video) adapted to the patient’s age and CHD, and address the main educational topics in youth with CHD (medical aspects, living with a CHD, nutrition, prophylaxis of endocarditis, social aspects, etc.). A CHD patient advocacy organisation (‘*Association Petit Cœur de Beurre*’) participated in the design of the educational content.

At the end of the 12-week intervention, participants attend an exit visit with the sports physician and health educator to review adherence, safety and completion of the rehabilitation programme before returning to routine clinical follow-up until the final 12-month assessment.

The secure digital platform brings together all the tools needed to deliver a home-based rehabilitation programme, with a collaborative approach: the age-appropriate patient interface provides the patient’s appointment calendar, videoconference meetings with each healthcare professional and the progress through the programme. The platform provides specific tools for healthcare professionals involved in the programme: secured medical electronic record, medical prescription and supervision of the programme, and recording of patient adherence in our trial.

### Outcomes

The primary endpoint is the change in the self-reported total score of the Pediatric Quality of Life Inventory V.4.0 Generic Core (PedsQL 4.0) questionnaire between baseline and 12-month follow-up in both groups. The PedsQL 4.0 questionnaire is a modular approach to measuring HRQoL in children with chronic diseases.^31^ This 23-item generic HRQoL questionnaire has four multidimensional scales (physical functioning, emotional functioning, social functioning and school functioning) and three summary scores ranging from 0 to 100 (total scale score, physical health summary and psychosocial health summary, including emotional, social and school functioning). Psychometric properties showed reliability, validity and responsiveness to clinical change over time,^32^ including the French and German versions of the PedsQL.^32 33^ Age-adapted versions of the PedsQL 4.0 self-questionnaire will be used (8–12, 13–17 and 18–25 years). The self-questionnaire will be completed independently by the participant, with assistance from a research assistant when required.

Secondary endpoints will include the change between baseline and 12-month follow-up in parent-reported HRQoL, neuropsychological outcomes, mental health outcomes and physical health outcomes. Safety and acceptability will also be assessed.

Parent-reported HRQoL will be assessed using the age-adapted versions of the generic PedsQL 4.0 proxy questionnaire, which will be completed independently by the parent or legal guardian of children aged 8–12 or 13–17 years, with assistance from a research assistant when required.

Neuropsychological and mental health outcome assessments will be performed at the investigating centre by a licensed neuropsychologist blinded to patients’ medical and surgical history as well as group assignment. Standardised neuropsychological tests will be administered by the neuropsychologist, whereas neuropsychological and mental health questionnaires will be completed by the participant and/or parent, according to the study protocol.

The following neuropsychological outcomes will be assessed:

- *Global cognitive outcomes:* using the Wechsler Intelligence Scale (WISC-V^34^ for children, WAIS-IV^35^ for adults), the following indices will be reported: full-scale IQ, verbal comprehension, visual-spatial abilities, working memory, fluid reasoning and processing speed. Each index has a mean (M) of 100 and a SD of 15.
- *Language, social cognition and memory:* we will use the NEPSY-II Child Neuropsychological Assessment battery, to assess language (comprehension of instructions), social cognition (facial emotion identification and theory of mind) and memory (designs) (M=10 and SD=3).^36^ Using the Wechsler Adult Memory Scale**,** selected verbal and visual memory subtests will be administered to adult participants.^37^
- *Executive functions:* we will use the *Wisconsin Card Sorting Test* to measure higher-order executive functioning including planning and cognitive flexibility.^38^ We will use the *Behavior Rating Inventory of Executive Function* (*BRIEF* for children and adolescents^39^ and *BRIEF-A* for adults^40^) which evaluates executive functions in daily-life for participants <18 years of age (parent report) and adults (self-report). A Global Executive Composite score will be calculated (M=50; SD=10).
- *Adaptive functioning:* the *Vineland-2* parent-questionnaire will be used to measure adaptive functioning and adaptive skills including social, communication, academic and daily-life independence. It will only be administered as a parent report for participants <18 years of age.^41^

The following mental health outcomes will be assessed:

- *Depression*: the *Multiscore Depression Inventory for Children* (*MDI-C*) scale will assess the child’s emotional world and more specifically to depression through eight dimensions: self-esteem, anxiety, sad mood, social introversion, pessimism, defiance, low energy and feelings of powerlessness.^42^ The *Beck’s Depression Inventory* (*BDI-2*) will be used for participants 18 years and older.^43^
- *Anxiety:* the *Revised Child Manifest Anxiety Scale R-CMAS (Child Manifest Anxiety Scale-Revised*), a self-administered questionnaire, will provide a more specific assessment of anxiety in its multiple expressions: worry/hypersensitivity, physiological anxiety and social concern or concentration.^44^ The *STAI State-Trait Anxiety Questionnaire* will be used for participants ≥18 years, providing a score of current and trait anxiety symptoms.^45^
- *Resilience:* the *Resilience Scale for Children* (*RS10*), a 10-item self-administered questionnaire will evaluate the child’s ability to cope and respond to life events.^46^ We will use the *Resilience Scale VK+* for participants ≥16 years old.^47^

*Physical health outcomes* will include clinical data (heart rate, blood pressure, body mass index), cardiopulmonary fitness, muscular strength and the level of physical activity. Cardiopulmonary fitness will be determined from the main CPET parameters (VO_2max_, ventilatory anaerobic threshold (VAT), ventilatory efficiency (VE/VCO2 slope), maximum oxygen pulse (VO_2max_/heart rate) and maximum workload), using a single harmonised cycle ergometer protocol previously described by our group (online supplemental document 1).^2 3 26^ The muscular strength will be measured using a handgrip dynamometer.^48 49^ The level of physical activity will be assessed by an accelerometer (Actigraph GT3X, Pensacola, Florida, USA). Accelerometry can be used to obtain daily sedentary time, daily physical activity times of light, moderate, vigorous and moderate-to-vigorous (MVPA) intensity, and number of steps per day.^50^ The level of physical activity is also measured by physical activity self-questionnaires (Physical Activity Questionnaire for Children (PAQ-C) for 8–13-year-old children, PAQ-A for 14–17-year-old adolescents and International Physical Activity Questionnaire – Short Form (IPAQ-SF) for patients ≥18 years old).^51–55^

*Safety outcomes* will be assessed in both groups and prospectively collected from patient enrolment to the final visit (12-month follow-up). Serious and non-serious adverse events will be analysed by the data safety and monitoring board members, blinded to the allocated group, to determine their relation to the intervention.

The *acceptability* of the neuro-cardiac rehabilitation programme to patients will be determined by adherence to the programme, compliance to each component of the programme (exercise training sessions, e-content viewing rate, etc.) and patient motivation. The adherence will be estimated by the participation rates (number of sessions delivered vs theoretical number of sessions), for the overall programme and each component of the programme.

Medical and surgical history will be reported in both groups, and socio-economic status of the patients and/or their families will be assessed at baseline.

All outcomes are summarised in online supplemental table 2.

### Study oversight

The study is sponsored by Bordeaux University Hospital, directed by a steering scientific committee (STC), supervised by a project manager (CJ), and supported by a local clinical research assistant in each investigating centre. The STC is directed by coordinating principal investigators (JC and PA), with the support of methodologists and project manager, and will make all decisions concerning the proper implementation of the protocol, oversee ethical aspects, and will keep up to date with the research progress, possible issues, and available results via the Data Methodology and Management Centre and coordinating investigator research centre. A Trial Management Team (TMT) was established for the trial for day-to-day operations. The TMT includes the coordinating principal investigators (PA and JC) and project manager (CJ). Patient and family advocacy group ‘*Petit Coeur de Beurre*’ are study partners in the design and communication of this study. Finally, a European consortium has been established for this study and is composed of principal investigators and scientific directors (JC and PA), the patient organisation (GM), as well as one key investigator in Belgium (SM) and Germany (AH).

### Patient and public involvement

The research questions and outcomes of this study were developed by considering challenges in patients with CHD. Patient association ‘Petit Coeur de Beurre’ was involved in the identification of key questions for this study. They will be involved in the dissemination of results to parents of children with CHD.

### Sample size

The primary outcome is the change in the self-reported HRQoL score with the PedsQL 4.0 instrument from baseline to 12-month follow-up. The minimal clinically important HRQoL difference was estimated from our previous controlled studies^56–58^ and randomised controlled trials using patient-reported outcomes as primary endpoints.^59 60^ Therefore, we hypothesised to observe an increase in the overall HRQoL score variation of 6±14 points (from 77.3 to 83.3 points) (over 100). With a 80% power, a bilateral alpha risk of 5% and potentially 10% loss to follow-up and/or missing data on the primary outcome, 96 patients need to be randomised to each group (N=192 total).

### Statistical analysis

All subjects enrolled will be included in the description of the population (baseline characteristics). An intention-to-treat (ITT) analysis will be used. To comply with the ITT principle, when at least one of the four HRQoL multidimensional scores (physical, emotional, social and school functioning) are missing at baseline or final assessment, multiple linear imputation will be implemented using a fully conditional specification method.^61^ A sensitivity analysis will be performed on complete data (patients with complete baseline and final assessment). A per-protocol analysis will be conducted, including all randomised subjects with no major protocol deviation, and defined as follows: 18-month maximum delay between baseline and final primary outcome assessment, patients in the intervention group who have successfully completed the neuro-cardiac rehabilitation, with at least 66% of the sessions (ie, participation in at least 16 out of 24 physical activity sessions and 16 out of 24 neuropsychological sessions, either Cogmed or the neuropsychological feedback), and for patients in the control group, a stable level of physical activity, defined as less than an increase of +15.6 min of daily MVPA time.^62^ Any rehabilitation or neuropsychological interventions received outside the study protocol during follow-up will be prospectively documented as concomitant care and considered when interpreting the study findings. The physical activity criterion was selected to minimise contamination bias related to increased physical activity potentially induced by participation in the QUALINEURO-REHAB study.

Baseline characteristics of the two groups will be reported using means and SD for continuous variables and with frequencies and percentages for categorical variables. Descriptive analyses will be overall and by group. Comparative analyses between groups will be performed systematically with adjustments on the randomisation stratification factor (country, age group). For each variable, missing data and reasons for it will be documented. For each outcome, the change between baseline and 12-month follow-up will be evaluated by a covariate analysis adjusted on the baseline value, age, sex and allocated intervention group. To assess the centre effect and the influence of other confounding factors, a mixed-effect model will be used considering the clinical site both as a random and then as a fixed variable and adjusting on confounding factors. The effect size will be estimated by the absolute difference of means and the value of Cohen’s d with their 95% CI.

Statistical analyses will be conducted by a biostatistician at the Methodology and Data Management Centre (USMR, Bordeaux University Hospital, France). All tests will be two-sided with a statistical significance set as 0.05, and analyses will be conducted using Statistical Analysis Systems V.7.13 (SAS).

### Ethics

The study will be conducted in compliance with the Good Clinical Practices protocol and Declaration of Helsinki principles. As required by European regulations, the trial protocol was first approved by the sponsor’s drawn National Ethics Committee (CPP Paris-III, number 2023-A00038-37), on 13 April 2023, and registered on Clinicaltrial.gov (number NCT05670132) on 4 January 2023. It was also approved by Ethics Committees in Germany (Technische Universitat Munchen, number 2023-276 S-KH, on 2 October 2023).

### Dissemination and data sharing

Dissemination of findings will be in the form of scientific articles in peer-reviewed journals and presentations at conferences. Any publication or communication will comply with the international recommendations: ‘Recommendations for the Conduct, Reporting, Editing, and Publication of Scholarly work in Medical Journals’ (http://www.icmje.org/recommendations). All participants will give written informed consent (online supplemental material 2) or assent to participate. The anonymised data to be collected in this study will be available within the manuscripts published. The Dissemination and data sharing section stating that participant data supporting the study findings will be available upon reasonable request, in accordance with ethical and legal requirements. In addition, the data sharing plan has been updated in the ClinicalTrials.gov registration to comply with *BMJ Open* and ICMJE requirements.

## Discussion

This protocol has presented the background and design for a European randomised controlled trial investigating the efficacy of a 12-week neurocardiac intervention for children, adolescents and young adults with CHD who underwent treatment in infancy. We will evaluate quality of life, neuropsychological and cardiovascular outcomes. The results from this trial will provide information on potential patient-specific factors associated with response to the intervention.

QUALINEURO-REHAB will combine, for the first time, cardiac rehabilitation and neuropsychological training for youth with CHD. This holistic approach stems from our previous research studies providing strong evidence that improvements in quality of life^26^ and neurodevelopmental outcomes^28^ are possible, yet need to be increased and generalised. Our current trial entails the whole definition of ‘rehabilitation’ per WHO guidelines as ‘the coordinated sum of activity and interventions required to ensure the best possible physical, mental, and social conditions so that patients may, by their own efforts, preserve or resume their proper place in society and lead an active life’.^63^ In CHD, the most frequent morbidities are neurodevelopmental sequelae and physical deconditioning. Together, these issues can cause exponential challenges in terms of academic achievement and social integration, mental health and cardiovascular outcomes, which in turn may substantially reduce quality of life. Timely rehabilitation of these issues is a public health priority in the care of individuals with CHD. If proven effective, holistic neurocardiac interventions could be implemented in a clinical outpatient practice for patients at increased neurodevelopmental, mental health and cardiovascular risk.

A key originality of QUALINEURO-REHAB is its hybrid model of care. The programme combines a digital platform for home-based rehabilitation with real-time appointments involving physicians, neuropsychologists, specialised nurses and adapted physical activity professionals. In addition, APA trainers will physically visit patients at home to support training and ensure adherence. This unique design aims to maximise accessibility, provide true multidisciplinary input and strengthen patient and family engagement. Nevertheless, adherence to such a comprehensive programme may still be challenging for some young people due to motivational, academic or family constraints. These aspects are not formally measured in the present protocol, but future research should complement quantitative outcomes with qualitative insights into participants’ experiences, barriers and facilitators to optimise long-term implementation.

Despite these limitations, this RCT is expected to provide robust evidence on the efficacy of a holistic neurocardiac intervention. Its findings could lay the foundation for broader integration of such programmes into paediatric cardiology rehabilitation practice across Europe.

## Supplementary material

10.1136/bmjopen-2026-120953 online supplemental file 1

10.1136/bmjopen-2026-120953 online supplemental file 2
