# Supplementary material for: Home-based neuro-cardiac rehabilitation in congenital heart disease: the QUALINEURO-REHAB trial study protocol
Source: BMJ Open. 2026 Oct 5;16(10):e120953. doi: 10.1136/bmjopen-2026-120953 (PMC13646947; doi:10.1136/bmjopen-2026-120953)
Supplement: Supplementary file 1 [file bmjopen-16-10-s001.pdf]

Supplementary Table 1. High-intensity interval training (HIIT) for the intervention group

Weeks	Steps	Warm-up intensity (%HR max)	Warm-up duration	Exercise intensity (%HR max)	Exercise duration	Active rest intensity (%HR max)	Active rest duration	Recovery intensity (%HR max)	Recovery duration	Total duration	Number of exercises
1	1	40 – 60%	5’	65 – 75 %	30’’	40 – 60%	2’	40 – 60%	5’	30’	8
2	2	40 – 60%	5’	75 – 90%	30’’	40 – 60%	2’	40 – 60%	5’	30’	8
3	3	40 – 60%	5’	75 – 90%	1’	40 – 60%	1’30	40 – 60%	5’	30’	8
4	3	40 – 60%	5’	75 – 90%	1’	40 – 60%	1’30	40 – 60%	5’	30’	8
5	4	40 – 60%	5’	75 – 90%	1’30	40 – 60%	1’	40 – 60%	5’	30’	8
6	4	40 – 60%	5’	75 – 90%	1’30	40 – 60%	1’	40 – 60%	5’	30’	8
7	4	40 – 60%	5’	75 – 90%	1’30	40 – 60%	1’	40 – 60%	5’	30’	8
8	4	40 – 60%	5’	75 – 90%	1’30	40 – 60%	1’	40 – 60%	5’	30’	8
9	4	40 – 60%	5’	75 – 90%	1’30	40 – 60%	1’	40 – 60%	5’	30’	8
10	4	40 – 60%	5’	75 – 90%	1’30	40 – 60%	1’	40 – 60%	5’	30’	8
11	4	40 – 60%	5’	75 – 90%	1’30	40 – 60%	1’	40 – 60%	5’	30’	8
12	4	40 – 60%	5’	75 – 90%	1’30	40 – 60%	1’	40 – 60%	5’	30’	8

Legend: HR, heart rate; max, maximum; 5’, five minutes; 30’’, thirty seconds.

Supplementary Table 2. Outcomes measures

**Primary outcome**
Health-related quality of life self-reported: Pediatric Quality of Life Inventory (PedsQL) self-questionnaire
**Secondary outcomes**
Neuropsychological outcomes
Global Cognitive Outcomes
Wechsler Intelligence Scale for Children (WISC-V) or Wechsler Adult Intelligence Scale (WAIS-IV)
Language, social cognition and memory
NEPSY-II Child Neuropsychological Assessment battery
Wechsler Adult Memory Scale
Executive functions
Wisconsin Card Sorting Test
Behavior Rating Inventory of Executive Function (BRIEF) for children (parent-reported) and BRIEF-A for adults (self-reported)
Adaptive functioning
Vineland-2 proxy-questionnaire (proxy-reported)
Mental health
Multiscore Depression Inventory for Children (MDI-C) or Beck Depression Inventory (BDI-2) for adults

Revised Child Manifest Anxiety Scale (R-CMAS) or State-Trait Anxiety Inventory for adults
Resilience Scale for Children (RS10) or Resilience for Adults VK+
Physical outcomes
CPET parameters
$VO2_{max}$ (maximum oxygen uptake)
VAT (ventilatory anaerobic threshold)
VE/VCO2 slope (ventilatory efficiency)
Maximum oxygen pulse
Maximum workload
Muscular strength: Handgrip test
Level of physical activity
Daily physical activity level measured by accelerometry (Accelerometer Actigraph GT3X): sedentary time, physical activity times of light, moderate, vigorous and moderate-to-vigorous (MVPA) intensity, steps per day
Physical Activity Questionnaire for Children (PAQ-C) for 8 to 13-year-old children, PAQ-A for 14 to 17-year-old adolescents and International Physical Activity Questionnaire – Short Form (IPAQ-SF) for adults
Clinical outcomes: NYHA functional class, blood pressure, body mass index (BMI) and medication

Health-related quality of life proxy-reported: PedsQL proxy-questionnaire (aged 8-17 years old)
Socio-economic status of the patient and/or the family (only at baseline)
Safety outcomes
Acceptability of the neuro-cardiac rehabilitation program

Legend: CPET, cardiopulmonary exercise test; NYHA, New York Heart Association; VCO2, carbon dioxide volume; VE, ventilation.

Supplementary Document 1. Cardiopulmonary exercise test protocol

Cardiopulmonary fitness will be evaluated using a unified pediatric Cardiopulmonary Exercise Test (CPET) protocol consistent with our previous similar studies.[3,26,64,65] Exercise laboratories for each center will follow a high-quality score rating ($\geq$ 10), according to the American Thoracic Society (ATS) and the American College of Chest Physicians (ACCP) statement on CPET,[66] employed adapted equipment and procedures: adapted pediatric and adult face masks, calibration of gas analyzers, utilization of breath-to-breath measurement software, 12-lead electrocardiogram equipment, and a pulse oximeter, mask leak assessment before and during exercise with monitoring of VO_2/watts ratio. Spirometry using a common gas device will be systematically performed before the exercise test with a flow-volume curve and measurement of forced expiratory volume in one second (FEV1), forced vital capacity (FVC), and the FEV1/FVC ratio (FEV1 %), with normalization to theoretical values.[67]

Pediatric and young adult participants will undergo a cycle ergometer to obtain a homogeneous incremental overall duration between 8 and 12 minutes, including a) 1-minute baseline; b) 3-minute warm-up (10-20 watts); c) fixed increments of 5-30 watts/min; d) pedaling rate of 60-80 revolutions/min; e) 3-minute active recovery; f) 2-minute rest. CPET was considered maximal when two of the following four criteria will be reached: (1) respiratory exchange ratio $\geq$ 1, (2) maximum heart rate > 85% of maximal age-predicted heart rate (220-age), (3) limit of the patient's tolerance despite verbal encouragement, and (4) plateau of VO_2 despite increasing exercise intensity.

The main parameter reflecting cardiopulmonary fitness used is the maximum oxygen uptake (VO_{2max}), an important marker of health.[68,69] In children, the VO_{2max} will be expressed in mL/Kg/min and in Z-score, from the recent pediatric CPET reference equations, derived from a nonlinear model using natural logarithms of VO_{2max}, height, and body mass index (BMI).[64,65] In adults, VO_{2max} will also be expressed in mL/Kg/min and as a percentage of the predicted reference value from Wasserman's linear equations.[70] Other CPET parameters will be collected, including the ventilatory anaerobic threshold (VAT) estimated using the V-slope method,[71] ventilatory efficiency (VE/VCO_2 slope), maximum oxygen pulse, and maximum workload. One investigator per center will interpret CPET, and in children, parameters will be expressed in Z-score from the same pediatric CPET reference equations.[64,65] In children, impaired VO_{2max} and VAT will be defined as Z-score < -1.64, corresponding to the 5th percentile for standard normal distribution.[64,65] In adults, $VO_{2max} \leq 80\%$ or VAT $\leq 50\%$ will indicate impaired cardiopulmonary fitness using reference equations.[70]

References

64. Amedro P, Matecki S, Pereira Dos Santos T, et al. Reference Values of Cardiopulmonary Exercise Test Parameters in the Contemporary Paediatric Population. *Sports Med Open* 2023;9:68.

65. Gavotto A, Mura T, Rhodes J, et al. Reference values of aerobic fitness in the contemporary paediatric population. *Eur J Prev Cardiol* 2023;30:820–9.

66. American Thoracic Society, American College of Chest Physicians. ATS/ACCP Statement on Cardiopulmonary Exercise Testing. *Am J Respir Crit Care Med* 2003;167:211–77.

67. Quanjer PH, Stanojevic S, Cole TJ, et al. Multi- ethnic reference values for spirometry for the 3–95- yr age range: the global lung function 2012 equations. *Eur Respir J* 2012;40:1324–43.

68. Raghuveer G, Hartz J, Lubans DR, et al. Cardiorespiratory Fitness in Youth: An Important Marker of Health: A Scientific Statement From the American Heart Association. *Circulation* 2020;142:e101–18.

69. García- Hermoso A, Ramírez- Vélez R, García- Alonso Y, et al. Association of Cardiorespiratory Fitness Levels During Youth With Health Risk Later in Life. *JAMA Pediatr* 2020;174:952.

70. Wasserman K, Hansen JE, Sue DY, et al. Principles of Exercise Testing and Interpretation. J *Cardiopulm Rehabil* 1987;7:189.

71. Beaver WL, Wasserman K, Whipp BJ. A new method for detecting anaerobic threshold by gas exchange. *J Appl Physiol* 1986;60:2020–7.
