# Supplementary material for: Home-based neuro-cardiac rehabilitation in congenital heart disease: the QUALINEURO-REHAB trial study protocol
Source: BMJ Open. 2026 Oct 5;16(10):e120953. doi: 10.1136/bmjopen-2026-120953 (PMC13646947; doi:10.1136/bmjopen-2026-120953)
Supplement: Supplementary file 2 [file bmjopen-16-10-s002.pdf]

NOTE D'INFORMATION

PARENT(S) OU TITULAIRE(S) DE L'AUTORITE PARENTALE

***Impact d'un programme de réhabilitation neuro-cardiaque sur la qualité de vie des enfants et jeunes adultes avec cardiopathie congénitale : l'essai multicentrique randomisé contrôlé* QUALINEURORÉHAB**

CHUBX 2021/66
Promoteur de la recherche : **CHU de Bordeaux - 12, rue Dubernat, 33404 Talence cedex**
Coordonnateur de l'étude : ***Pr Pascal AMEDRO***

Madame, Monsieur,

Votre enfant a été vu en consultation par un cardiopédiatre pour le suivi de sa malformation cardiaque appelée « cardiopathie congénitale ».

Nous vous proposons de faire participer votre enfant à un protocole de recherche intitulé : « **Impact d'un programme de réhabilitation neuro-cardiaque sur la qualité de vie des enfants et des jeunes adultes avec cardiopathie congénitale : l'essai multicentrique randomisé contrôlé QUALINEURORÉHAB** », promu par le CHU de Bordeaux et coordonnée par le Pr Pascal Amedro. Nous vous demandons de lire attentivement cette note d'information avant de prendre votre décision pour faire participer votre enfant à cette étude. Ce document a pour but de vous expliquer pourquoi nous vous proposons cette étude pour votre enfant, comment celle-ci va se dérouler et quels en sont les risques potentiels. Vous disposez d'un délai de réflexion pour prendre votre décision.

Si vous acceptez que votre enfant participe à cette recherche, et s'il le souhaite lui aussi, vous pourrez à tout moment durant l'essai vous adresser à votre médecin investigateur pour lui poser toutes questions complémentaires.

Sa participation est entièrement volontaire. Si vous ne désirez pas que votre enfant prenne part à cette recherche, ou s'il ne veut pas y participer, il continuera à bénéficier de la meilleure prise en charge médicale possible, conformément aux connaissances actuelles.

Quel est l'objectif de la recherche ?

Les anomalies cardiaques sont les premières causes de malformation à la naissance. Presque 1 enfant sur 100 naît avec une malformation cardiaque. Les progrès de la médecine en cardiologie, anesthésie-réanimation et chirurgie cardiaque ont considérablement amélioré le pronostic des cardiopathies congénitales sur les dernières décennies. Aujourd'hui, il s'agit donc de « vivre » plutôt que « survivre » avec une cardiopathie congénitale.
De nombreux patients ayant une cardiopathie congénitale ont une activité physique insuffisante, alors qu'elle pourrait être souvent autorisée et encouragée par les médecins. De plus, le suivi, depuis le diagnostic jusqu'aux soins à l'âge adulte, s'est adapté et se concentre maintenant sur l'évaluation et l'amélioration de la qualité de vie des personnes. Dans cette perspective, deux grands enjeux sanitaires ont récemment émergé : la promotion de l'activité physique et la prévention des problèmes à long terme.
Cette étude propose donc d'évaluer l'impact d'un programme de prévention et de réadaptation "global", associant une intervention neuropsychologique et de l'activité physique sur la qualité de vie des enfants, des adolescents et des jeunes adultes ayant une cardiopathie congénitale par rapport à leur prise en charge habituelle.

Qu'est-ce que le programme de réhabilitation neuro-cardiaque ?

Dans la pratique courante, les programmes de réhabilitation cardiaques se déroulent durant 3 semaines en milieu hospitalier, dans des centres de « réadaptation cardio-vasculaire ».

Concernant les difficultés neuropsychologiques (des difficultés de réalisation de tâches quotidiennes, des difficultés de concentration dans le quotidien, à l'école ou dans la vie professionnelle, ou des difficultés d'interactions sociales, …) et en santé mentale, elles sont aujourd'hui peu dépistées, et leur prise en charge n'est pas généralisée.

Notre programme, associant une intervention neuropsychologique et de l'activité physique, se déroule « à la maison » et a été construit spécifiquement pour que les jeunes patients et leur famille adhèrent à la réhabilitation, afin de favoriser l'autonomie des patients et d'intégrer ce programme dans leur vie quotidienne.

Qu'est-ce que la qualité de vie ?

La qualité de vie c'est à la fois le bien-être physique et mental, l'absence de douleur, la possibilité d'avoir des amis, de faire des études, de pratiquer un sport, d'avoir des loisirs, etc. Des questionnaires ont été créés pour mesurer la qualité de vie à l'aide de scores, permettant de mesurer le retentissement d'une maladie sur la vie quotidienne. Ces questionnaires sont adaptés à l'âge du patient.

Comment se déroulera cette étude ?

Afin de valider nos résultats, cette étude se déroulera dans plusieurs établissements hospitaliers en Europe (France, Belgique et Allemagne). Elle est coordonnée par le service de cardiologie congénitale pédiatrique et adulte du CHU de Bordeaux.

Au total, 192 patients ayant une cardiopathie congénitale et âgés de 8 à 25 ans participeront à cette étude. Ils seront affectés dans un groupe par tirage au sort (c'est ce qu'on appelle la randomisation) :

- Les patients affectés dans le groupe « intervention » participeront au programme de réhabilitation neurocardiaque, à domicile.
- Les patients affectés dans le groupe contrôle auront leur suivi habituel pendant un an.

Qui peut participer ?

Pour participer à cette étude, les patients doivent être âgés de 8 à 25 ans et porteurs d'une malformation cardiaque congénitale opérée durant leur 1ère année de vie.

Votre enfant ne pourra pas participer s'il présente, entre autres, des troubles psychiatriques sévères, une hypertension ou des troubles du rythme cardiaque non contrôlés, ou s'il a déjà réalisé une réhabilitation cardiaque ou cognitive dans les 6 mois précédents l'inclusion. Cette liste n'est pas exhaustive et l'équipe médicale se chargera de vérifier que votre enfant répond bien à l'ensemble des critères d'inclusion et de non-inclusion pour valider sa participation.

Quels sont les bénéfices attendus ?

Avant d'analyser nos résultats, nous ne pouvons donc pas, à ce stade, affirmer que notre programme entraine un bénéfice pour les patients. Si les résultats sont positifs, cette étude pourrait permettre aux enfants et jeunes adultes avec une cardiopathie d'adhérer à un programme de réhabilitation, de l'intégrer dans leur vie quotidienne et ainsi d'avoir un *"esprit sain dans un corps sain"*.

De plus elle pourrait avoir un impact majeur en matière de santé publique dans l'organisation et la prise en charge des patients atteints de cardiopathie congénitale.

Enfin, le développement de ce programme global de traitement combiné pourrait servir de modèle pour le suivi d'autres maladies chroniques impliquant un déconditionnement physique et une altération de la santé mentale.

CHU BDX CENTRE HOSPITALIER UNIVERSITAIRE BORDEAUX

Quels sont les risques prévisibles ?

La participation de votre enfant à cette recherche ne présente pas de risque prévisible. Sur les données connues et rapportées actuellement, les programmes de réhabilitation, sous contrôle médical, ne présentent pas plus de risque que les activités de la vie quotidienne.

Quelle sera la durée de participation de l'enfant ?

La durée de participation de votre enfant sera au maximum de 1 an.

Quel est le calendrier de suivi de votre enfant dans le cadre de cette étude ?

Si vous acceptez que votre enfant participe à cette étude, et s'il remplit toutes les conditions requises pour y participer, le calendrier de suivi sera le suivant :

• **Visite d'inclusion pouvant être associée à sa visite de suivi annuelle** : Celle-ci consiste en une consultation en présentielle avec un cardiologue ou cardiopédiatre qui vous expliquera le protocole et répondra à vos questions.

Si vous êtes d'accord pour que votre enfant participe le consentement de participation devra être signé par vous et le médecin. Votre enfant aura ensuite :

- Les examens manquants de son suivi de routine le cas échéant,
- Un auto-questionnaire sur la qualité de vie et un sur l'activité physique,
- Une évaluation de sa force musculaire (pression d'une main),
- Une évaluation neuropsychologique comprenant différents tests ludiques et questionnaires.

A la fin de cette visite, un petit appareil enregistrant son activité (actimètre) sera positionné au niveau de sa taille et effectuera un enregistrement sur une période de 7 jours. Ce dispositif devra être retourné au centre à l'issue de ces 7 jours.

• **Tirage au sort :** une fois l'ensemble des examens d'inclusion réalisés et validés, le tirage au sort pourra être réalisé afin de déterminer la prise en charge de votre enfant. Le résultat vous sera communiqué par téléphone à partir du 7ème jour après la visite d'inclusion et au plus tard 14 jours après celle-ci:

- **Si votre enfant fait partie du groupe « intervention »**, il suivra le programme de réhabilitation neurocardiaque, à domicile, pendant 12 semaines.
- **Si votre enfant fait partie du groupe « contrôle »**, il suivra les recommandations de son cardiologue.

• **Visite de fin d'étude à douze mois pouvant être associée à sa visite de suivi annuelle** : Cette visite peut être couplée avec le bilan annuel de votre enfant, réalisée par le cardiopédiatre ou le cardiologue et correspond à une consultation, un examen clinique, un électrocardiogramme (ECG), une échocardiographie et un test d'effort cardio-respiratoire. Lors de la visite de fin d'étude votre enfant aura donc en plus des examens cités précédemment, une évaluation neuropsychologique, une mesure de la force musculaire (pression d'une main), un questionnaire d'évaluation de la qualité de vie, un questionnaire d'activité physique, une évaluation de l'activité physique (port d'un actimètre pendant 7 jours avant la consultation). Vous aurez aussi de nouveau à remplir des questionnaires sur le fonctionnement de l'enfant dans sa vie quotidienne. Dans l'éventualité où les examens ne pourraient être programmés et se dérouler dans la même journée, cette dernière visite pourrait être réalisée en deux temps sur une période de 8 jours.

Le programme de réhabilitation « QUALINEUROREHAB » se déroule à domicile, sur trois mois, en deux phases. Il vous sera demandé de vous connecter à une plateforme numérique sécurisée, la plateforme QUALIREHAB, dont vous recevrez un mail pour la création de votre mot de passe personnel à la suite de l'élaboration de l'identifiant de votre enfant par l'équipe investigatrice. Cette plateforme est développée par la société Anamnèse©, sur des serveurs certifiés « hébergeur de données de santé ». Seuls les personnels soignants et d'investigation prenant en charge votre enfant auront accès à vos données.

A) Il commence par une période d'initiation multidisciplinaire comprenant des téléconsultations avec le médecin du sport, le neuropsychologue et l'infirmière spécialisée. Les téléconsultations seront organisées au cours de la première semaine du programme, le même jour ou non, mais devront toujours commencer

par la consultation avec le médecin du sport. Tous ces rendez-vous se déroulent via la plateforme QUALIREHAB. L'évaluation initiale multidisciplinaire dure 2 à 3 heures au cours de laquelle :

- ✓ Le programme de rééducation physique sera présenté et les besoins spécifiques des patients évoqués,
- ✓ Les composantes et l'organisation du programme d'entrainement neurocognitif seront introduites et les modalités d'utilisation de la plateforme de tests discutées,
- ✓ Des objectifs éducatifs sont déterminés.

B) <u>Il se poursuit par le programme d'entraînement à domicile, qui comporte trois volets</u> :

(1) Le volet « activité physique » consiste en deux séances d'activités physiques adaptées par semaine pendant 12 semaines, encadrées par un enseignant en activité physique adapté (APA) ou équivalent. Une séance sur 2 aura lieu en présentiel et l'autre par vidéoconférence via la plateforme QUALIREHAB. Les deux séances d'entraînement suivent le même schéma : 30 minutes de vélo fixé à un home trainer avec une intensité progressivement croissante ; puis 30 minutes d'activité physique ; au choix de votre enfant et déterminée conjointement avec l'enseignant APA.

(2) Le volet « neuropsychologique » consiste en 2 séances hebdomadaires, de 25 minutes chacune, d'entraînement cognitif pour le patient en autonomie à l'aide d'une tablette ou d'un ordinateur via le logiciel Cogmed®, logiciel d'entrainement neurocognitif. Toutes les activités de formation seront supervisées par des psychologues formés à cette recherche, qui se rendront disponible en cas de questions. S'en suivra une séance hebdomadaire de 30-45 minutes avec un(e) psychologue agréé(e) afin de faire un point sur les entrainements neuropsychologiques de la semaine ainsi que sur des stratégies pour améliorer les fonctions exécutives de votre enfant (régulation des émotions, capacités à se concentrer) dans la vie quotidienne.

(3) Le volet « éducation thérapeutique » comporte un accès pendant toute la durée du programme à des documents et vidéos, via la plateforme QUALIREHAB, vous permettant ainsi que votre enfant, de mieux connaitre sa cardiopathie, vous donner des outils pour bien vivre avec, et l'aider à avoir la meilleur santé physique et mentale. Ces supports seront sélectionnés spécifiquement pour vous et votre enfant par une infirmière en éducation thérapeutique au début du programme.

Ce programme nécessite un investissement et une assiduité importants car il implique 4 séances hebdomadaires (2 séances d'activité physique et 2 neuropsychologiques) pendant 12 semaines. Il nécessite une connexion internet et un matériel informatique (PC, tablette ou smartphone) pour vous connecter avec les professionnels de santé lors des séances en vidéoconférence et avoir accès au programme d'éducation thérapeutique.

Quels sont vos droits en tant que participant à cette recherche ?

Vous pouvez arrêter la participation de votre enfant à tout moment de l'essai sans justification, sans conséquence sur la suite de son traitement ni la qualité des soins qui lui seront fournis et sans conséquence sur la relation avec votre médecin. Il pourra être suivi par la même équipe médicale. La participation de votre enfant à cette recherche n'engendrera aucun frais supplémentaire pour vous. Si vous acceptez que votre enfant participe au présent projet, tous les frais liés à l'étude seront pris en charge par le promoteur.

De quelle protection bénéficiez-vous dans le cadre de cette recherche ?

Le promoteur qui assure la gestion, le financement de tous les frais et la responsabilité de ce projet de recherche est le Centre Hospitalier Universitaire (CHU) de Bordeaux situé au 12 rude Dubernat, 33004 Talence Cedex, France. Le CHU de Bordeaux a pris toutes les dispositions prévues par la loi 2012-300 du 5 mars 2012 relatives aux recherches impliquant la personne humaine et à la loi Informatique et Liberté du 6 janvier 1978 modifié. Le CHU de Bordeaux devant assumer l'indemnisation des éventuelles conséquences dommageables de la recherche biomédicale pour la personne qui s'y prête, a souscrit une assurance de recherches biomédicales, conformément à la législation en vigueur.

Lorsque la responsabilité du promoteur n'est pas engagée, les participants peuvent être indemnisés auprès de l'ONIAM, (Office National d'Indemnisation des Accidents Médicaux, 36, Avenue du Général de Gaulle, 93175 BAGNOLET Cedex, N° Vert : 0800 779 887) dont la mission concerne l'indemnisation des victimes d'aléas thérapeutiques. Les modalités de ce protocole ont été soumises pour avis au Comité de Protection des Personnes (CPP). Ce comité a pour mission de vérifier la pertinence scientifique des recherches impliquant la personne humaine, les conditions requises pour la protection et le respect des participants. Le Comité de Protection des Personnes a rendu un avis délibératif favorable à la réalisation de cette recherche. L'ANSM a été informée du déroulement de cette recherche.

Aspect confidentiel des données

Le dossier médical de votre enfant restera confidentiel et ne pourra être consulté que sous la responsabilité du médecin s'occupant de son traitement ainsi que par les autorités de santé et par des personnes dûment mandatées par le promoteur de l'essai et soumises au secret professionnel.

Dans le cadre de la recherche interventionnelle à contraintes minimes impliquant la personne humaine, à laquelle il vous est proposé de faire participer votre enfant, un traitement automatisé et pseudo-anonymisé de ses données personnelles va être mis en œuvre pour permettre d'analyser les résultats de la recherche au regard de l'objectif de cette dernière qui vous a été présentée. A cette fin, les données médicales concernant votre enfant seront transmises au Promoteur de la recherche. Ces données seront identifiées par un numéro de code et/ou vos initiales. Ces données pourront également, dans des conditions assurant leur confidentialité, être transmises aux autorités de santé françaises.

Conformément aux dispositions de loi relative à l'informatique et aux libertés (loi n° 78-17 du 6 janvier 1978 modifiée par les lois n° 94-548 du 1er Juillet 1994, n° 2002-303 du 4 mars 2002, et 2004-801 du 6 août 2004) vous disposez d'un droit d'accès (article 39) et de rectification (article 40) aux informations qui concernent votre enfant. Vous pouvez également, pour des motifs légitimes, vous opposer au traitement des données concernant votre enfant. Ces droits s'exercent auprès du médecin en charge de la recherche qui seul connaît l'identité de votre enfant. Vous pouvez également accéder directement ou par l'intermédiaire d'un médecin de votre choix à l'ensemble des données médicales de votre enfant en application des dispositions de l'article L 1111-7 du Code de la Santé Publique. Les informations concernant l'identité de votre enfant seront tenues confidentielles par le médecin investigateur.

De plus si vous le souhaitez, vous serez informé, à votre demande, des résultats globaux de l'essai par le médecin investigateur.

A qui devez-vous vous adresser en cas de questions ou de problèmes?

Votre contact dans l'étude
Dr/Pr…………………...-Investigateur<br>Service de cardiologie pédiatrique<br>Téléphone :………………………………..

CHU BDX CENTRE HOSPITALIER UNIVERSITAIRE BORDEAUX

FORMULAIRE DE CONSENTEMENT

PARENT OU TITULAIRE DE L'AUTORITE PARENTALE

Impact d'un programme de réhabilitation neuro-cardiaque sur la qualité de vie des enfants et jeunes adultes avec cardiopathie congénitale: l'essai multicentrique randomisé contrôlé QUALINEUROREHAB

CHUBX 2021/66
Promoteur de la recherche : **CHU de Bordeaux - 12, rue Dubernat, 33404 Talence cedex**
Coordonnateur de l'étude : ***Pr Pascal AMEDRO***

Nous soussignés, Mme……………………………………………………………………………………….(*nom, prénom) et* M ……………………………………………………………………………… (*nom, prénom),* parents *(ou titulaires de l'autorité parentale, lien de parenté avec l'enfant)* de ……………………………………………………………………… *(nom, prénom de l'enfant)* certifions avoir lu et compris la note d'information qui nous a été remise.
Nous avons eu la possibilité de poser toutes les questions que nous souhaitions au Pr/Dr……………………………………………..*(nom, prénom)* qui nous a expliqué la nature, les objectifs, les risques potentiels et les contraintes liées à la participation de notre enfant à cette recherche.
Nous connaissons la possibilité qui nous est réservée d'interrompre la participation de notre enfant à cette recherche à tout moment sans avoir à justifier notre décision et nous ferons notre possible pour en informer l'investigateur qui suit notre enfant dans la recherche. Cela ne remettra naturellement pas en cause la qualité des soins ultérieurs.

Nous avons eu l'assurance que les décisions qui s'imposent pour la santé de notre enfant seront prises à tout moment, conformément à l'état actuel des connaissances médicales.

Nous avons pris connaissance que cette recherche a reçu l'avis favorable du Comité de Protection des Personnes *Ile de France III en date du / / et* a fait l'objet d'une information auprès de l'ANSM.
Le CHU de Bordeaux a signé un engagement de conformité à la « Méthodologie de référence » (MR-001) en application des dispositions de l'article 54 alinéa 5 de la loi du 6 janvier 1978 modifiée relative à l'information, aux fichiers et aux libertés.

Le Promoteur de la recherche (CHU de Bordeaux, 12 rue Dubernat, 33404 Talence Cedex) a souscrit une assurance de responsabilité civile en cas de préjudice auprès de la compagnie LLOYD'S INSURANCE COMPANY SA par l'intermédiaire de la société de courtage BEAH SAS dont l'adresse est 16-18 rue de Londres, 75009 Paris (tel : 04 82 25 01 62) sous le n°B1339CTLICNWL22-54.

Nous acceptons que les personnes qui collaborent à cette recherche ou qui sont mandatées par le Promoteur, ainsi qu'éventuellement le représentant des Autorités de Santé, aient accès à l'information dans le respect le plus strict de la confidentialité.
Nous acceptons que les données enregistrées à l'occasion de cette recherche, sous forme pseudonymisée (codées), puissent faire l'objet d'un traitement informatisé sous la responsabilité du Promoteur. Nous sommes informés, qu'à l'issue de cette recherche, ces données pourront être utilisées à d'autres fins de recherche (nouvelles analyses statistiques) et nous avons été informés de notre droit de nous y opposer.
Nous avons bien noté que, conformément au Règlement Général sur la Protection des Données (RGPD) et aux dispositions de la loi relative à l'informatique, aux fichiers et aux libertés, nous disposons d'un droit d'accès, de rectification, de suppression, de limitation du traitement, à la portabilité des données, d'opposition et de retrait. Ces droits s'exercent auprès de l'investigateur qui suit notre enfant dans le cadre de cette recherche et qui connaît son identité.
Notre consentement ne décharge en rien l'investigateur et le Promoteur de la recherche de leurs responsabilités à l'égard de notre enfant. Notre enfant conserve tous les droits garantis par la loi.
Les résultats globaux de la recherche nous seront communiqués directement, si nous le souhaitons, conformément à la loi du 4 mars 2002 relative aux droits des malades et à la qualité du système de santé.

Ayant disposé d'un temps de réflexion suffisant avant de prendre notre décision, nous acceptons librement et volontairement que notre enfant participe à la recherche QUALINEURORÉHAB
Nous acceptons librement et volontairement que les données cliniques de notre enfant puissent être conservées et utilisées à d'autres fins de recherche (nouvelles analyses statistiques) ❒ ***oui*** ❒ ***non***

Nous acceptons librement et volontairement que notre médecin traitant/pédiatre soit informé de la participation de notre enfant et que les comptes rendus lui soit transmis ❒ ***oui*** ❒ ***non***

Nous pourrons à tout moment demander des informations complémentaires à l'investigateur qui a proposé à notre enfant de participer à cette recherche, n° téléphone :

Votre enfant a été informé(e) de sa participation à la recherche et ne s'y oppose pas.

Nom du premier parent (ou premier tuteur) : ....

Signature du premier parent (ou premier tuteur) : Date |__|__| |__|__| |__|__|__|__|

Nom du deuxième parent (ou deuxième tuteur) : ....

Signature du deuxième parent (ou deuxième tuteur) : Date |__|__| |__|__| |__|__|__|__|

Nom de l'investigateur : ....

Signature de l'investigateur : Date |__|__| |__|__| |__|__|__|__|
