# Supplementary material for: Home-based neuro-cardiac rehabilitation in congenital heart disease: the QUALINEURO-REHAB trial study protocol
Source: BMJ Open. 2026 Oct 5;16(10):e120953. doi: 10.1136/bmjopen-2026-120953 (PMC13646947; doi:10.1136/bmjopen-2026-120953)
Supplement: Supplementary file 3 [file bmjopen-2026-120953.reviewer_comments.pdf]

PEER REVIEW HISTORY

BMJ Open publishes all reviews undertaken for accepted manuscripts. Reviewers are asked to complete a checklist review form and are provided with free text boxes to elaborate on their assessment. These free text comments are reproduced below.

ARTICLE DETAILS

Title (Provisional)

Home-based neuro-cardiac rehabilitation in congenital heart disease: The QUALINEURO-REHAB trial study protocol

Authors

Calderon , Johanna; Martins, Sylvie; Hager, Alfred; Cornuault, Lauriane; Jore, Cecile; Andrianoely, Mathieu; Picot, Marie- Christine; Francis, Florence; Saillour, Florence; KRET, Marion; Huby, Anne Cécile; Audie, Marion; Duboue, Pierre-Marie; Hubrechts, Jelena; De La Villeon, Gregoire; Vincenti, Marie; Marguin, Gaelle; Desmure, Gwladys; Hock, Julia; Schmitz, David; Bonnet, Damien; Moniotte, Stephane; Guillaumont, Sophie; Amedro, Pascal; STUDY GROUP, QUALINEURO REHAB

VERSION 1 - REVIEW

**Reviewer**	**1**
**Name**	**Andrews, Jennifer**
**Affiliation**	**University of Arizona, Pediatrics**
**Date**	**22-Jun-2026**
**COI**	**None**

Thank you for the opportunity to review your manuscript. I believe the executive function deficits in individuals who are not affected severely enough to receive services in school are in the most need, which your protocol would address.

Below are some comments regarding your manuscript but I have some minor concerns with your approach.

Study Design – What is the association of echo and ECG with the trial? You should state that the trial itself is 12 weeks in the design section. What do the participants do after that and their 12 month follow-up? Also for those in the control arm, do you give them the opportunity to participate after their 12 months? Note that you describe the physical exam in detail but not the neuropsych

Table 1 – your days numbering is confusing – you are screening people up to 42 days prior to their visit? What is the rationale for that?

Study Population – your age stratification and N should be listed in this section – also are you controlling for activity status and changes in the participants?

Trial Procedures – please be more explicit about your randomization method. Are the sessions live? If live, separate patient/parent or combined? Are you providing the bikes to the participants? What is a home trainer?

Outcomes – you note patient involvement in study design. I think it would be incredibly valuable to do a short structured interview about why they feel their HRQoL is better or not and what about the program is valuable.

Overall the manuscript could benefit from some points of additional detail and shifting of information around.

VERSION 1 - AUTHOR RESPONSE

Reviewer 1 comments (Dr Jennifer Andrews, University of Arizona)

Comments to the Author:
Thank you for the opportunity to review your manuscript. I believe the executive function deficits in individuals who are not affected severely enough to receive services in school are in the most need, which your protocol would address. Below are some comments regarding your manuscript but I have some minor concerns with your approach.

1) "Study Design – What is the association of echo and ECG with the trial? You should state that the trial itself is 12 weeks in the design section. What do the participants do after that and their 12-month follow-up? Also, for those in the control arm, do you give them the opportunity to participate after their 12 months? Note that you describe the physical exam in detail but not the neuropsych."

<u>Response:</u> We thank the reviewer for these helpful comments. We have clarified the Study Design section to explicitly state that the intervention itself lasts 12 weeks, followed by usual clinical care until the 12-month outcome assessment. We also clarified that ECG and echocardiography are part of the routine cardiology evaluation performed before enrolment to document cardiovascular status, confirm eligibility, and ensure safe participation in the physical rehabilitation component of the intervention.

After the 12-week intervention, participants return to standard clinical follow-up, with no additional supervised study intervention before the 12-month assessment. Participants in the control arm continue standard care throughout the study; no crossover to the intervention is planned after 12 months because the efficacy of this program has not yet been established. However, any clinically indicated rehabilitation or neuropsychological care may be offered according to usual practice in each participating center.

Finally, the neuropsychological outcomes section (the neuropsychological intervention is described on page 8 (trial procedures), and neuropsychological outcomes are all detailed on pages 11-12).

2) Table 1 – your days numbering is confusing – you are screening people up to 42 days prior to their visit? What is the rationale for that?

Response: Thank you for pointing this out. The screening window of up to 42 days reflects routine clinical practice across participating centers. In several centers, the cardiology consultation, electrocardiogram, echocardiography, and cardiopulmonary exercise test cannot always be scheduled on the same day because of local organizational constraints, particularly

the availability of CPET. Consequently, these routine clinical assessments may be spread over approximately 4 to 6 weeks before enrolment. We have clarified this rationale in the legend of Table 1 to improve readability.

This was clarified as follows: “The screening period extends up to 42 days before inclusion to accommodate routine clinical practice across participating centers, where the cardiology consultation, ECG, echocardiography, and cardiopulmonary exercise test may be scheduled over several visits because of local logistical constraints.”

3) Study Population – your age stratification and N should be listed in this section – also are you controlling for activity status and changes in the participants?

Response: Age stratification is included in trial procedures along with all stratification criteria. The total N required for this trial is included in the Statistical Section as part of the statistical power calculations.

Finally, physical activity will be objectively monitored by accelerometry and complemented by validated self-reported questionnaires at baseline and 12 months.

4) Trial Procedures – please be more explicit about your randomization method. Are the sessions live? If live, separate patient/parent or combined? Are you providing the bikes to the participants? What is a home trainer?

Response: We have expanded the description of the randomization procedure by specifying that allocation is computer-generated, stratified by country and age group, with concealed allocation. We also clarified that all rehabilitation sessions are delivered live, either through videoconferencing or home visits depending on the intervention component. Finally, we clarified that bicycles are provided to participants throughout the intervention and that the "home trainer" refers to an indoor stationary trainer allowing participants to use the bicycle safely at home.

5) Outcomes – you note patient involvement in study design. I think it would be incredibly valuable to do a short structured interview about why they feel their HRQoL is better or not and what about the program is valuable.

Response: We appreciate this valuable suggestion. Although structured qualitative interviews are not included in the current protocol, acceptability of the intervention is assessed through predefined acceptability outcomes, including adherence, compliance and participant motivation. We also acknowledge in the Discussion that future studies should incorporate qualitative interviews to better understand participants' experiences and perceived benefits of the intervention (page 18).

6) Overall the manuscript could benefit from some points of additional detail and shifting of information around.

Response: We thank this reviewer for their suggestions. We have provided minor changes requested and have offered clarification on some points raised.

VERSION 2 - REVIEW

**Reviewer**	**1**
**Name**	**Andrews, Jennifer**
**Affiliation**	**University of Arizona, Pediatrics**
**Date**	**03-Aug-2026**

COI

Thank you for the revisions. I apologize if my previous concerns were unclear. As written, the manuscript is challenging to follow from a replicability standpoint. Yes the reader could probably piece together what is happening from the text, outcomes, and tables but it requires significant effort. I am uncertain if the headers you selected are required but there is sometimes a mismatch between the header and the content and what i would have expected to find is described elsewhere.

The following are still concerns from a reproducibility perspective (which I believe is key for a protocol manuscript)

1. You provide details regarding usual care that is NOT part of your study design and should therefore not be included but in the subsequent sentence you indicate that “each participant will undergo a similar physical” which is not defined. Are they undergoing all the tests listed for usual care again at enrollment and exit? If yes – why would you require these be done within 6 weeks prior to enrollment if they will be repeated. Your criteria state frequently ECG or CPET but the text reads “and.”

2. I do not see that you added the bike will be provided.

3. You indicate that they may receive these services as needed – that should then exclude them from the control group or warrant an early exit.

4. You have elected not to make any changes to the EF intervention description which remains very unclear to me and instead refer me to the outcomes which are tests and patient/parent report scales.

a. You are doing 25 minute Cog training – twice or twice weekly?

i. You do not define what is involved in executive function training or guidance training despite doing so in the exercise training (HIIT and free)

ii. What is entailed in the neurocognitive booklets and are they scaled for participant age?

b. You are doing 12 weeks of 1 hour session for patient and parent separately or combined?

c. You discuss things that are age appropriate solely with respect to the digital platform layout and not the exercise programs or the cognitive programs

d. You do not clearly describe the exit visit it is only presumed in outcomes and from the table.

e. The BRIEF is not available as self-report under age 11 but that is not stated. The Vineland questionnaire is solely parent report and that is also not stated.

f. Why are you not using the BRIEF2?

g. You do not clearly specify which of the tests in the outcomes are practitioner administered.

While I understand you do not want to be repetitive, your decision on where to place specifics makes the protocol challenging to follow.

VERSION 2 - AUTHOR RESPONSE

Reviewer 1, R1: *"You provide details regarding usual care that is NOT part of your study design and should therefore not be included but in the subsequent sentence you indicate that "each participant will undergo a similar physical" which is not defined. Are they undergoing all the tests listed for usual care again at enrollment and exit? If yes – why would you require these be done within 6 weeks prior to enrollment if they will be repeated. Your criteria state frequently ECG or CPET but the text reads "and.""*

Response: We have revised the *Study Design* section to clearly distinguish the screening procedures from the study-specific assessments. Screening procedures may be performed up to 42 days before enrolment because, in routine clinical practice across participating centers, the cardiology consultation, physical examination, ECG, echocardiography, and CPET are not always scheduled on the same day and may be spread over several visits due to local logistical constraints, particularly CPET availability. These routine clinical investigations are reviewed to confirm eligibility and ensure safe participation in the intervention. Following enrolment, all participants undergo standardized baseline study assessments before randomization, and the same research assessments are repeated at the 12-month follow-up visit. We also revised the wording regarding arrhythmias to clarify that uncontrolled arrhythmias detected on ECG and/or CPET constitute an exclusion criterion, consistent with the eligibility criteria.

2. I do not see that you added the bike will be provided.

Response: We apologize that this clarification was not clear in the previous version of the manuscript. The manuscript now explicitly states in the *Cardiac Rehabilitation* section that a bicycle and home trainer are provided to each participant in the intervention group for the duration of the 12-week intervention.

3. You indicate that they may receive these services as needed – that should then exclude them from the control group or warrant an early exit.

Response: We thank the reviewer for this important comment. We agree that this statement referred to routine clinical care rather than the study design and could therefore be misleading. We have removed this sentence from the *Study Design* section. Participants allocated to the control group receive standard care throughout the study, with no crossover to the QUALINEURO-REHAB intervention. To further address potential contamination bias, we clarified the *Statistical Analysis* section by specifying that any rehabilitation or neuropsychological interventions received outside the study protocol during follow-up will be prospectively documented. In addition, the per-protocol analysis includes a predefined criterion for physical activity in the control group (stable MVPA level), to minimize contamination bias related to increased physical activity potentially induced by participation in the study.

4. You have elected not to make any changes to the EF intervention description which remains very unclear to me and instead refer me to the outcomes which are tests and

patient/parent report scales.

Response: We thank the reviewer for this comment. We agree that the description of the executive function intervention required further clarification. Accordingly, we substantially expanded the *Neuropsychological Intervention* section (pages 9–10) to better describe the intervention content, objectives, delivery, and materials. Specifically, we now explain: (i) the executive function domains targeted by the Cogmed Working Memory program; (ii) the structure and content of the computerized training exercises; (iii) the organization of the weekly neuropsychological guidance sessions; (iv) the adaptation of the intervention and educational materials according to age group; and (v) the content and purpose of the neurocognitive booklets provided to participants and parents. We believe these revisions substantially improve the reproducibility of the neuropsychological intervention.

a. *You are doing 25-minute Cog training – twice or twice weekly?*

Response: Participants complete two 25-minute Cogmed sessions per week (approximately 50 minutes/week total).

i. *You do not define what is involved in executive function training or guidance training despite doing so in the exercise training (HIIT and free)*

Response: We have substantially expanded this description on pages 9–10 to clearly define both the executive function training and the neuropsychological guidance sessions.

ii. What is entailed in the neurocognitive booklets and are they scaled for participant age?

Response: We have clarified this in detail on pages 9-10. Neurocognitive booklets are adapted to participant age (8-12 years, 13-17 years and 18+). The contents follow the same pattern, only the content and topics are adapted to what is relevant and developmentally appropriate.

b. You are doing 12 weeks of 1 hour session for patient and parent separately or combined?

Response: Participants (parents of minors and adults with CHD) are doing 1 hour session during 12 weeks. Children <18 years only do Cogmed and their parents do the neuropsychological follow-up sessions. Adults with CHD do both, Cogmed and the neuropsychological follow-up.

c. You discuss things that are age appropriate solely with respect to the digital platform layout and not the exercise programs or the cognitive programs

Response: We thank the reviewer for this comment. We have clarified that both the cognitive and physical components of the intervention are age appropriate. The neuropsychological intervention and educational materials are tailored to three predefined age groups. Although the structure of the HIIT protocol is standardized, its intensity is individually prescribed from each participant's baseline CPET using heart rate targets, thereby accounting for age-related physiological differences and individual exercise capacity. In addition, the individualized exercise training component is adapted to each participant's age and functional abilities.

d. You do not clearly describe the exit visit it is only presumed in outcomes and from the table.

Response: We have clarified the intervention schedule by explicitly referring readers to Table 1, which summarizes the initiation visit (Week 1), the 12-week intervention, the exit visit at the end of Week 12, and the final 12-month follow-up assessment. We also revised the legend of Table 1 to clarify that the intervention starts with the sports physician consultation during the initiation week and ends with the sports physician exit consultation during the final week of the rehabilitation program.

e. The BRIEF is not available as self-report under age 11 but that is not stated. The Vineland questionnaire is solely parent report and that is also not stated.

Response: The BRIEF is only given to parents of children <18 years. The BRIEF-A is given to adults. The Vineland is only given to parents of children <18 years.

f. Why are you not using the BRIEF2?

Response: We are using the BRIEF instead of the BRIEF-2 because that was the only version available in France and Germany at the time of study implementation. We had described this in our manuscript (page 12). We have added "as a parent report" for the Vineland (page 12).

g. You do not clearly specify which of the tests in the outcomes are practitioner administered.

Response: We thank the reviewer for this helpful comment. We have clarified the administration of the outcome assessments throughout the *Outcomes* section. Specifically, we now distinguish between self-reported questionnaires, parent-reported questionnaires, practitioner-administered neuropsychological assessments, and clinical outcome assessments. We also clarified that the PedsQL™ self-questionnaire and proxy questionnaire are completed independently by the participant and the parent/legal guardian, respectively, with assistance from a research assistant when required. Standardized neuropsychological tests are administered by a licensed neuropsychologist, whereas patient- and parent-reported questionnaires are completed independently by participants or parents, as appropriate, with assistance from a research assistant when required. We also clarified the administration procedures for the primary and secondary outcome assessments throughout the Outcomes section.
